# Head-to-head comparison of [^11^C]methionine PET, [^11^C]choline PET, and 4-dimensional CT as second-line scans for detection of parathyroid adenomas in primary hyperparathyroidism

**DOI:** 10.1007/s00259-023-06488-7

**Published:** 2023-11-17

**Authors:** Milou E. Noltes, Schelto Kruijff, Auke P. A. Appelman, Liesbeth Jansen, Wouter T. Zandee, Thera P. Links, Bettien M. van Hemel, Hugo M. Schouw, Rudi A. J. O. Dierckx, Anne Brecht Francken, Wendy Kelder, Anouk van der Hoorn, Adrienne H. Brouwers

**Affiliations:** 1 Department of Nuclear Medicine and Molecular Imaging, University Medical Center Groningen, University of Groningen, Groningen, the Netherlands; 2 Department of Surgical Oncology, University Medical Center Groningen, University of Groningen, Groningen, the Netherlands; 3 Department of Surgery, Martini Hospital Groningen, Groningen, the Netherlands; 4 Department of Molecular Medicine and Surgery, Karolinska Institutet, Stockholm, Sweden; 5 Department of Radiology, University Medical Center Groningen, University of Groningen, Groningen, the Netherlands; 6 Division of Endocrinology, Department of Internal Medicine, University Medical Center Groningen, University of Groningen, Groningen, the Netherlands; 7 Department of Pathology, University Medical Center Groningen, University of Groningen, Groningen, the Netherlands; 8 Department of Surgical Oncology, Isala, Zwolle, the Netherlands

**Keywords:** Primary hyperparathyroidism, Imaging, Positron emission tomography (PET), Computed tomography (CT), Sensitivity, Parathyroid

## Abstract

**Purpose:**

Accurate preoperative localization is imperative to guide surgery in primary hyperparathyroidism (pHPT). It remains unclear which second-line imaging technique is most effective after negative first-line imaging. In this study, we compare the diagnostic effectiveness of [^11^C]methionine PET/CT, [^11^C]choline PET/CT, and four dimensional (4D)-CT head-to-head in patients with pHPT, to explore which of these imaging techniques to use as a second-line scan.

**Methods:**

We conducted a powered, prospective, blinded cohort study in patients with biochemically proven pHPT and prior negative or discordant first-line imaging consisting of ultrasonography and ^99m^Tc-sestamibi. All patients underwent [^11^C]methionine PET/CT, [^11^C]choline PET/CT, and 4D-CT. At first, all scans were interpreted by a nuclear medicine physician, and a radiologist who were blinded from patient data and all imaging results. Next, a non-blinded scan reading was performed. The scan results were correlated with surgical and histopathological findings. Serum calcium values at least 6 months after surgery were used as gold standard for curation of HPT.

**Results:**

A total of 32 patients were included in the study. With blinded evaluation, [^11^C]choline PET/CT was positive in 28 patients (88%), [^11^C]methionine PET/CT in 23 (72%), and 4D-CT in 15 patients (47%), respectively. In total, 30 patients have undergone surgery and 32 parathyroid lesions were histologically confirmed as parathyroid adenomas. Based on the blinded evaluation, lesion-based sensitivity of [^11^C]choline PET/CT, [^11^C]methionine PET/CT, and 4D-CT was respectively 85%, 67%, and 39%. The sensitivity of [^11^C]choline PET/CT differed significantly from that of [^11^C]methionine PET/CT and 4D-CT (*p* = 0.031 and *p* < 0.0005, respectively).

**Conclusion:**

In the setting of pHPT with negative first-line imaging, [^11^C]choline PET/CT is superior to [^11^C]methionine PET/CT and 4D-CT in localizing parathyroid adenomas, allowing correct localization in 85% of adenomas. Further studies are needed to determine cost–benefit and efficacy of these scans, including the timing of these scans as first- or second-line imaging techniques.

**Supplementary Information:**

The online version contains supplementary material available at 10.1007/s00259-023-06488-7.

## Introduction

Primary hyperparathyroidism (pHPT) is a common endocrine disorder, with the highest incidence in post-menopausal women [1]. The incidence of pHPT is estimated to be 65.5 per 100,000 person-years among women and 24.7 per 100,000 person-years among men [1].

Surgery is the only curative treatment for patients with pHPT, and is associated with a decreased risk of fractures and nephrolithiasis, and long-term improvement in quality of life [2–4]. As in 90% of patients, only one parathyroid adenoma is present, a minimally invasive parathyroidectomy (MIP) is the preferred surgical approach [5]. In comparison to a bilateral neck exploration, a MIP is associated with less postoperative morbidity with similar cure rates [6, 7]. In order to perform a MIP, accurate preoperative imaging is essential. Worldwide, the currently advised first-line preoperative imaging approach consists of cervical ultrasonography (cUS) combined with ^99m^Tc-methoxyisobutylisonitrile-single-photon emission computed tomography/computed tomography ([^99m^Tc]Tc-MIBI-SPECT/CT) [8–10]. This combination of scans has proven to be the most cost-effective strategy and is widely available [9]. However, both modalities have limited sensitivity [10–13]. Unsuccessful preoperative localization of the adenoma would subsequently mandate traditional bilateral neck exploration, exposing both recurrent laryngeal nerves, risking postoperative hypoparathyroidism and increased scarring in the neck.

Recently, there has been increased interest in the use of positron emission tomography/computed tomography (PET/CT) for parathyroid imaging. Both [^11^C]methionine PET/CT and [^18^F/^11^C]choline PET/CT can be used as second-line scans after prior negative first-line localization [14–16]. Furthermore, four-dimensional CT (4D-CT) can be used for the localization of parathyroid adenomas [17]. 4D-CT consists of serial CT scans acquired at different stages of enhancement and washout of contrast media in the parathyroid glands, and its neighboring organs and tissues [18]. The sensitivity of [^11^C]methionine PET/CT is reported between 60 and 86% [19–21], of [^18^F/^11^C]choline PET/CT between 87 and 96% [15, 21–25] and of 4D-CT between 80 and 90% [13, 26, 27]. However, no studies are available comparing these three techniques directly. Therefore, it remains unclear which of these imaging techniques is most effective after negative first-line imaging. Consequently, in routine daily care, the preoperative imaging workup is managed in a heterogeneous manner [28].

There is a clear clinical need for more accurate localization of parathyroid adenomas preoperatively when first-line imaging fails. This will enable more targeted, less extensive, and more effective surgical strategies. Therefore, the main aim of this study was to directly compare the sensitivity of [^11^C]methionine PET/CT with [^11^C]choline PET/CT. Furthermore, the study aimed to assess the sensitivity of 4D-CT, and which of these three imaging techniques to use as a second-line scan in the setting of pHPT.

## Methods

### Study design and patients

This is a single-center prospective, blinded interventional study primarily designed to compare the sensitivity of [^11^C]methionine PET/CT with [^11^C]choline PET/CT and secondarily 4D-CT in patients with biochemically proven pHPT and prior negative, inconclusive or discordant first-line imaging consisting of cUS and [^99m^Tc]Tc-MIBI^−^SPECT/CT in a tertiary teaching referral hospital in the Netherlands between March 2019 and August 2022.

Patients eligible for inclusion were (i) ≥ 18 years old, (ii) had biochemically confirmed pHPT (defined as elevated serum calcium adjusted for albumin in the presence of an elevated or inappropriately normal PTH, or normal adjusted total calcium and normal ionized calcium levels along with elevated PTH on at least two occasions [29]), (iii) underwent a [^99m^Tc]Tc-MIBI-SPECT/CT and cUS with negative or discordant results, (iv) had an indication for parathyroidectomy, and (v) were eligible for surgery. Patients were excluded (i) if they were known to have a germline mutation predisposing for multiple gland disease, (ii) if an alternative diagnosis (e.g., parathyroid carcinoma) was suspected before surgery, if they had (iii) a previous negative neck exploration for pHPT, (iv) persistent pHPT after previous negative neck exploration, if they had (v) an eGFR < 30 ml/min × 1.73 m^2^, (vi) or an allergy for iodinated contrast, or (vii) if they were pregnant.

First-line imaging results were categorized as negative when they failed to visualize any parathyroid adenoma, or when read as inconclusive, not convincingly showing an adenoma. First-line imaging results were classified as discordant if a potential adenoma was identified on only one imaging modality (e.g., negative [^99m^Tc]Tc-MIBI-SPECT/CT and positive cUS) or if both imaging modalities showed an adenoma but in different locations. Subsequently, all patients in this study underwent [^11^C]methionine PET/CT, [^11^C]choline PET/CT, and 4D-CT. The main outcome was the sensitivity of [^11^C]methionine PET/CT and [^11^C]choline PET/CT. Secondary outcomes were the sensitivity of 4D-CT and the positive predictive value (PPV) of [^11^C]methionine PET/CT, [^11^C]choline PET/CT and 4D-CT.

The study was registered at the Netherlands Trial Register (NTR7423) and approved by the Research Ethics Board of the University Medical Center Groningen, The Netherlands (METc 2018/330). This study was performed in line with the principles of the Declaration of Helsinki. Written informed consent was obtained from all subjects.

### Scan interpretation

For the methods of all imaging techniques, refer to the Supplemental Material in Online Resource 1. All patients underwent all three imaging techniques ([^11^C]methionine PET/CT, [^11^C]choline PET/CT, and 4D-CT).

All scans ([^11^C]methionine PET/CT, [^11^C]choline PET/CT, and 4D-CT) were blinded from patient data and previous imaging modalities, and subsequently separately evaluated by one dedicated nuclear medicine physician (A. H. B.) for the PET/CT scans and one of two dedicated radiologists (A. P. A. A. or A. H.) for the 4D-CT. During the blind evaluation, no clinical or other imaging results were available to the readers. Hereafter, the radiologist and nuclear medicine physician came to a non-blinded consensus regarding the localization of the parathyroid adenoma. In this setting, the readers could make use of all available clinical and imaging data, including the original imaging reports of all scans. Scan outcomes were classified as negative (not, or not convincingly showing an adenoma), or as positive (suspected adenoma with its respective location). The location of the suspected adenoma was described in relation to the position of the thyroid midline/trachea. This resulted in the following possible locations: right cranial, right caudal, left cranial, left caudal, or ectopic (e.g., mandibular angle; paratracheal, paratracheal-esophageal, or mediastinal).

### Surgery

A MIP was performed via a lateral keyhole incision of 2.5 cm medial of the sternocleidomastoid muscle and lateral of the anterior strap muscles. When needed, a bilateral neck exploration was performed via a Kocher incision (usually 3 cm) over the thyroid parallel to the skin folds. The aim of surgery was to identify and remove the parathyroid adenoma, if possible concordant with preoperative imaging (based on the non-blinded consensus reading). Final localization of the adenoma during surgery was retrieved from the anatomic description in the surgical report. The pathology report was reviewed for the final diagnosis. Cure was defined as normal serum albumin-corrected calcium level (< 2.55 mmol/L) 6 months after surgery [30].

Preoperative adenoma localization of both the blinded and non-blinded scan evaluations were defined as true positive, false positive, and false negative, depending on both the surgical and pathology report and serum calcium levels at 6 months post-surgery. We do not report on true negative results since the surgeon did not systematically explore all potential embryological locations to exclude a parathyroid adenoma. A true positive lesion was defined as pathological proven adenoma localized to the correct quadrant on pre-operative imaging. A false positive lesion was defined as a suspected adenoma localized to the incorrect quadrant, or a suspected lesion in a quadrant while surgically no pathological parathyroid gland was found in this quadrant. Lastly, a false negative lesion was classified as removal of a pathological proven parathyroid adenoma during surgery, although the scan had not demonstrated the presence of hyperactive parathyroid tissue in this quadrant.

### Statistical analysis

A power analysis, before we executed this prospective study, using the method of Connor et al. was performed on the main outcome (sensitivity of [^11^C]methionine PET/CT of 72%[19] and of [^11^C]choline PET/CT of 97%[25]), and with these sensitivities as our aim, sample size calculation with a two-sided significance level of 0.05 and power of 80%, revealed a needed requirement (higher bound) of 29 patients [31].

Data were analyzed using descriptive statistics. Continuous variables with normal distribution or abnormal distribution are displayed by mean ± standard deviation (SD) or median with interquartile range (IQR), respectively. Sensitivity and PPV were calculated lesion-based. To compare the sensitivity across the different imaging techniques, the McNemar’s test was performed [32]. A two-sided *p*-value < 0.05 was considered statistically significant. Statistical analyses were performed using IBM SPSS Statistics version 27.0 (IBM Corp., Armonk, NY, USA).

## Results

### Study group

A total of 32 patients were included, of whom two were replaced according to protocol because these patients did eventually not want to undergo parathyroid surgery. These patients were included in the scan interpretation analyses (*n* = 32 patients), but could not be evaluated in the surgical lesion-based analysis (*n* = 30 patients). In total, twenty-four patients were female (75%) and the median age was 64.0 (IQR 16.5) years (Table 1). Median preoperative corrected calcium levels were 2.70 (IQR 0.17) mmol/L and PTH levels 10.3 (IQR 5.8) pmol/L. All included patients (100%) were hypercalcemic, and no patient was normocalcemic.

**Table 1:** Patients’ baseline characteristics

Characteristics	Total cohort (*n* = 32)
Gender, *n* (%)	
Female	24 (75%)
Age, years	
Median (IQR)	64.0 (16.5)
Body mass index, kg/m^2^	
Median (IQR)	26.7 (8.74)
Preoperative albumin-corrected calcium (mmol/L)	
Median (IQR)	2.70 (0.17)
Preoperative PTH (pmol/L)	
Median (IQR)	10.3 (5.8)

*IQR* interquartile range; reference range albumin-corrected calcium = 2.10–2.55 mmol/L; reference range PTH =  < 8.7 pmol/L

### Scan interpretation

**Fig. 1:**
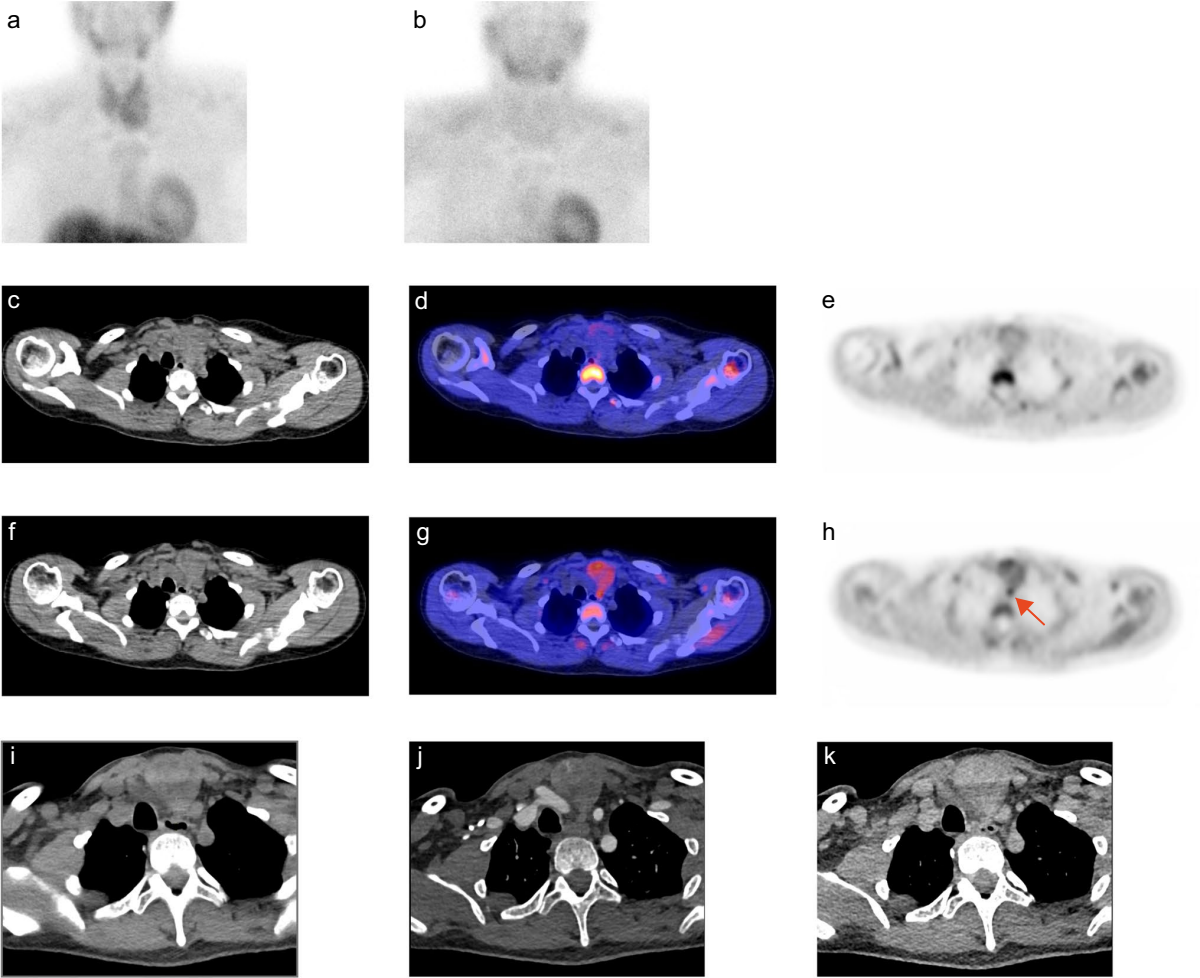
Preoperative parathyroid imaging of a representative patient. Planar anterior image of the neck with [^99m^Tc]Tc-MIBI (**a** early, **b** late) does not localize the parathyroid adenoma. The [^11^C]methionine PET/CT (**c** ldCT image, **d** fused PET/CT image, and **e** PET only image) also does not clearly locate a parathyroid adenoma (negative at both the blinded and non-blinded interpretation). On the [^11^C]choline PET/CT (**f** ldCT image, **g** fused PET/CT image, and **h** PET only image), a lesion suspicious for a parathyroid adenoma was shown caudally to the left thyroid lobe (red arrow in h). During parathyroidectomy, a parathyroid adenoma (0.9 cm and 0.18 g at pathology) was removed from this exact location. The 4D-CT scan (**i** nonenhanced phase, **j** arterial phase, **k** venous phase) did not evidently show the classic characteristic contrast enhancement pattern of parathyroid adenoma and was therefore declared as negative based on the blinded and non-blinded interpretation

**Fig. 2:**
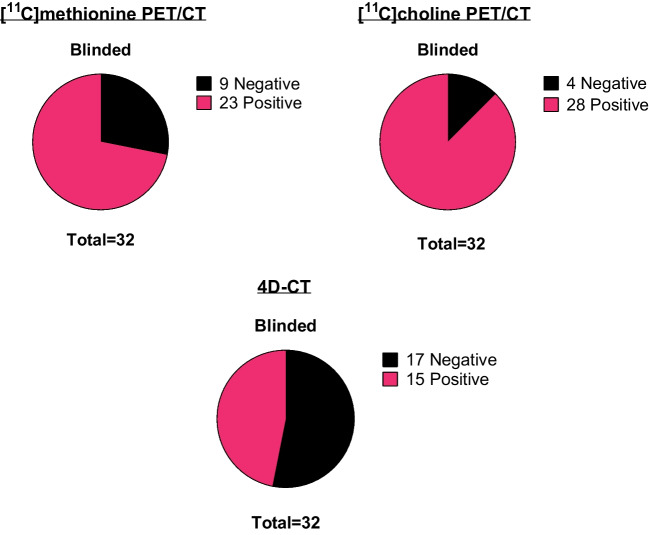
Preoperative parathyroid scan interpretation of [^11^C]methionine PET/CT, [^11^C]choline PET/CT, and 4D-CT with blinded scan interpretation (patient-based, *n* = 32)

**Fig. 3:**
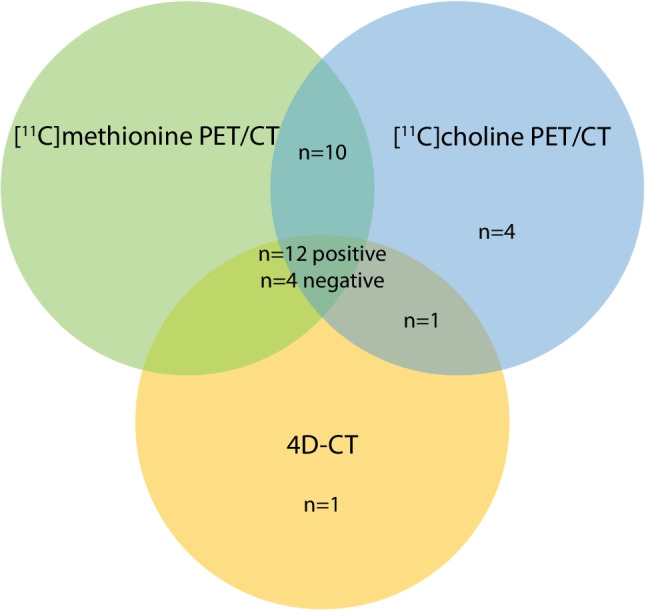
Overlap between scan interpretation of [^11^C]methionine PET/CT, [^11^C]choline PET/CT, and 4D-CT with blinded scan interpretation (patient-based, *n* = 32). In *n* = 12 patients, all scans were positive, and in *n* = 4 patients, all scans were negative

For results of the first-line imaging techniques, refer to the Supplemental Material in Online Resource 1. For results of the non-blinded scan interpretation, refer to the Supplemental Material in Online Resource 1. Figure 1 shows the images of a representative patient. When the scans were blinded from patient data and previous imaging modalities, [^11^C]methionine PET/CT was positive in 23 patients (72%) and identified 23 lesions suspicious for a parathyroid adenoma (Fig. 2). For [^11^C]choline PET/CT, this was the case in 28 patients (88%) identifying 32 lesions, whereas 4D-CT was positive in 15 patients (47%) identifying 16 lesions. For the overlap between the three imaging techniques, refer to Fig. 3.

Interestingly, 14 patients (44%) underwent a [^18^F]choline PET/CT prior to inclusion in our study. The [^18^F]choline PET/CT was negative in 12 patients and inconclusive in two patients, whereas at the blinded scan interpretation, [^11^C]methionine PET/CT, [^11^C]choline PET/CT, and 4D-CT could identify a lesion suspicious for a parathyroid adenoma in 10 (71%), 12 (86%), and 7 (58%) of these patients. Figures 6 and 7 in Online Resource 1 display examples of such cases with a previous negative [^18^F]choline PET/CT.

### Surgery

Thirty patients (94%) underwent a surgical procedure and were included for the final analysis of the diagnostic effectiveness. Twenty-five patients (83%) underwent a focused parathyroidectomy, and five patients (17%) a bilateral exploration. In total, 32 parathyroid lesions were histologically confirmed as parathyroid adenomas. Median weight and diameter of parathyroid adenomas were 0.35 g (IQR 0.46 g) and 13.0 mm in largest diameter (IQR 6 mm). Three patients had multi-gland disease (*n* = 6 lesions) presumably not associated with multiple endocrine neoplasia syndrome. In 26 patients, one parathyroid adenoma was removed, and in one patient, no parathyroid tissue was removed during surgery.

In 29 patients (97%), 6-month follow-up calcium levels were available and biochemical cure was confirmed in 27/29 patients (93%). One patient had follow-up shorter than 6 months because he died due to a cardiac arrest; however, he was cured on day one postoperatively. In the two patients in whom biochemical cure was not achieved 6 months after surgery, in one patient, no parathyroid tissue was removed during surgery. The [^11^C]methionine PET/CT, [^11^C]choline PET/CT, and 4D-CT suggested a lesion caudal to the left submandibular gland (level IB). During surgery, exploration of the area of the submandibular gland was performed, but the presence of parathyroid tissue could not be confirmed by the pathologist. This patient was diagnosed with persistent hyperparathyroidism. In the lesion-based analysis, it was classified as both a false positive and a false negative lesion. The other non-cured patient was a patient with multiple gland disease. During surgery, two parathyroid glands were removed and were both confirmed as parathyroid hyperplasia. This patient was normocalcemic on postoperative day one; however, unfortunately, 6 months after surgery, the patient became hypercalcemic again. All scans in this patient were false negative. In the lesion-based analysis, they were classified as two false negative lesions. For additional results of the surgical procedure, refer to the Supplemental Material in Online Resource 1.

### Accuracy of study imaging modalities

For results of the non-blinded scan interpretation, refer to the Supplemental Material in Online Resource 1. In the patients with multi-gland disease (*n* = 3 patients and *n* = 6 lesions), [^11^C]methionine PET/CT and 4D-CT were true positive in one lesion (17%), while [^11^C]choline PET/CT was true positive in four lesions (75%) at the blinded scan interpretation.

**Fig. 4:**
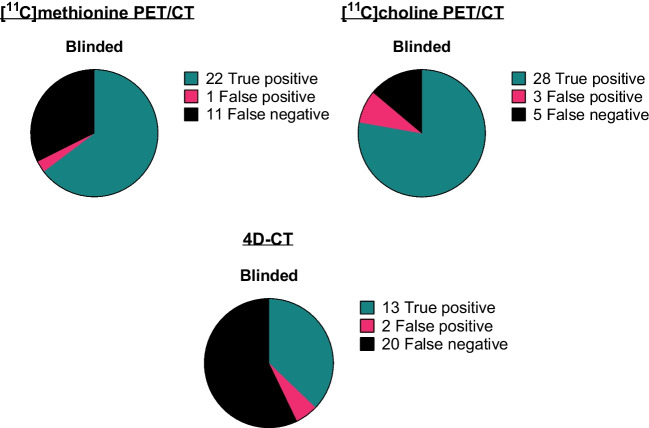
Effectiveness of the three studied imaging modalities for parathyroid adenoma localization with blinded scan interpretation (lesion-based). Adenoma localization on preoperative imaging was correlated with intraoperative surgeon localization and histopathological findings (reference standard). The total number of reference standard lesions (true positive + false negative) consists of *n* = 32 pathology-proven parathyroid lesions and one patient with a negative exploration (*n* = 1 lesion) in *n* = 30 operated patients

After blinded scan interpretation, [^11^C]methionine PET/CT resulted in a lesion-based sensitivity and PPV of 67% (95% CI: 48% to 82%) and 96% (95% CI: 76% to 100%), [^11^C]choline PET/CT in a sensitivity of 85% (95% CI: 68% to 95%) and PPV of 90% (95% CI: 73% to 98%), and 4D-CT in 39% (95% CI: 23% to 58%) and 87% (95% CI: 58% to 98%), respectively (Fig. 4). The sensitivity of [^11^C]methionine PET/CT was higher when the scans were blindly interpreted, whereas for 4D-CT, it was evidently lower and for [^11^C]choline PET/CT, it was similar (Table 2 and Table 1 in Online Resource 1). For the overlap between scan interpretation and histological results, refer to Fig. 4 in the Supplemental Material in Online Resource 1.

**Table 2:** Final lesion-based outcome with blinded scan interpretation for the n = 30 operated patients*

	Blinded scan interpretation				
	True positive	False positive	False negative	Sensitivity (95% CI)	PPV (95% CI)
[^11^C]Methionine PET/CT	22	1	11	67% (48–82%)	96% (76–100%)
[^11^C]Choline PET/CT	28	3	5	85% (68–95%)	90% (73–98%)
4D-CT	13	2	20	39% (23–58%)	87% (58–98%)

*CI* confidence interval, *PPV* positive predictive value

^*^Total consists of 32 pathology-proven parathyroid lesions and one patient with a negative exploration

The sensitivity of [^11^C]choline PET/CT differed significantly from [^11^C]methionine PET/CT and 4D-CT for the blinded interpretations (*p* = 0.031 and *p* < 0.0005, respectively). The sensitivity of [^11^C]methionine PET/CT and also 4D-CT differed significantly for the blinded interpretations (*p* = 0.012).

## Discussion

This study prospectively compared the diagnostic accuracy of [^11^C]methionine PET/CT, [^11^C]choline PET/CT, and 4D-CT head-to-head in patients with pHPT. Our results show that [^11^C]choline PET/CT is superior to [^11^C]methionine PET/CT and 4D-CT in localizing parathyroid adenomas.

Interestingly, the sensitivity for [^11^C]methionine PET/CT imaging decreased when the scans were interpreted non-blindly, as compared to the blinded interpretation. Contrarywise, the sensitivity and PPV for 4D-CT imaging evidently increased during the non-blinded scan interpretation. This finding may be the result of intra-observer variability and can be used as an additional argument to primarily employ ([^11^C]choline) PET/CT over 4D-CT imaging, since PET imaging initially resulted in more accurate findings, whereas for 4D-CT, more information from other imaging modalities was required. The blinded scan interpretation more closely resembles current practice, as these three imaging modalities will likely not all be performed in a patient with pHPT. Even though in the blinded scan interpretation [^11^C]methionine was superior to 4D-CT, one may consider performing 4D-CT after a negative [^11^C]choline PET/CT to obtain more information from an anatomical perspective (in addition to the ultrasound).

Several studies found that [^11^C]methionine PET/CT, [^11^C/^18^F]choline PET/CT, and 4D-CT are able to localize parathyroid lesions when conventional imaging failed to do so [13, 19–21, 23–25, 27, 33, 34]. A recent study prospectively comparing [^11^C]methionine PET/CT and [^18^F]choline PET/CT in 26 patients that underwent parathyroid surgery found that in patients with negative or inconclusive [^99m^Tc]Tc-MIBI, [^18^F]choline PET/CT had a significant better performance than [^11^C]methionine PET/CT for the detection of pathological parathyroid tissue [21]. A recent meta-analysis showed that [^18^F]choline PET/CT had a higher sensitivity than 4D-CT, although only one of the included studies performed a head-to-head comparison [35]. This meta-analysis therefore also concluded that more comparative studies on the diagnostic performance of these imaging methods are needed. To the best of our knowledge, this is the first study comparing the three imaging techniques prospectively in the same patient for the preoperative detection and localization of hyperfunctioning parathyroid tissue.

The uptake of [^11^C]methionine most likely depends on expression and activity of amino-acid transporters such as LAT1, and secondarily on its incorporation in the protein pre-pro-PTH [21]. [^11^C]methionine is therefore closely related to the synthesis and excretion of PTH. Choline-labeled radiopharmaceuticals, thanks to their positive electric charge, enter through a membrane transporter and accumulate in the mitochondria of both oxyphilic and chief cells. Furthermore, in the chief cells, choline is also phosphorylated by choline-kinase, which is overexpressed in patients with HPT, and used as a component of cell membranes [21]. This double uptake of choline-labeled radiopharmaceuticals mechanism could represent the advantage of choline PET/CT to methionine PET/CT [36, 37].

The choice for [^11^C]choline rather than for 4D-CT, next to its superior diagnostic effectiveness, may also be supported by the consideration that 4D-CT generally involves a higher radiation exposure than [^11^C]choline PET/CT [24, 38–42]. Additionally, there is slight chance of an allergic reaction to the iodinated contrast of the 4D-CT scan. However, the main advantage of 4D-CT lies in its wide availability in most clinical environments, its detailed anatomical information, the absence of the need for tracer production, and due to more accessible patient preparation, no uptake time (and related waiting time) of tracer is necessary. The performance of 4D-CT in this study is relatively poor compared to previous studies (sensitivity of 75 to 80%) [43–45]. We hypothesize that this is due to the highly selected group of patients with previous negative imaging results.

Interestingly, in 12 out of 14 patients in whom [^18^F]choline PET/CT was negative or inconclusive before enrollment in the study, [^11^C]choline PET/CT could identify a lesion suspicious of a hyperfunctioning parathyroid gland. Therefore, in this study, [^11^C]choline PET/CT seemed to be superior to [^18^F]choline PET/CT for localization of parathyroid lesions. The cause of this finding unfortunately remains speculative. [^18^F]Choline, a tracer comparable to [^11^C]choline, is a more widely available tracer because of its longer half-life (110 min). We hypothesize that the improved identification with [^11^C]choline is mainly caused by differences in scan protocols between centers. For [^11^C]choline, we always employ an uptake time of 20 min and a scan duration of 10 min, after optimization of the protocol [46]. Most centers that use [^18^F]choline scan after 2, 30, and 60 min with a maximum scan duration of 5 min [25, 47–51]. Also, differences in biodistribution of the tracers cannot be ruled out. Since a direct comparison between the two choline tracers will likely not be performed in patients, only ex vivo experiments using both choline tracers may shed more light on this issue.

Based on the current results, [^11^C]choline PET/CT is the preferred choice for second-line imaging in our clinical setting. We are aware that in other clinics this tracer has drawbacks. These drawbacks consist of the need for an on-site cyclotron for the production of the short-lived isotope [^11^C] (20 min half-life), and a GMP radiopharmacy facility for the production of [^11^C]choline. Although [^11^C]choline PET/CT has shown superior results, data from large cohorts and on cost-efficacy are not currently available. Therefore, more research is warranted into the cost–benefit and efficacy of the three studied imaging techniques, also in the setting as first-line imaging techniques.

In this study, we chose to exclude patients with a germline mutation predisposing for multiple gland disease to maintain homogeneity within the study group. Therefore, we cannot elaborate on the diagnostic performance of these imaging modalities in this subgroup of patients. We did previously study the diagnostic performance of [^11^C]choline PET/CT in a small group of patients with multiple endocrine neoplasia and found a sensitivity of 67% [25]. However, more research is still warranted into additional preoperative imaging in these patients.

A limitation of this study could be that the preoperative work-up (including cUS and [^99m^Tc]Tc-MIBI-SPECT/CT) prior to inclusion in the study was partly performed heterogeneously at outside institutions and reviewed by different nuclear medicine physicians, reflecting the real world setting. However, after inclusion, the work-up was homogenously performed as all imaging techniques were performed in the same hospital according to standardized protocols and reviewed by experts in the field. Also, the external [^99m^Tc]Tc-MIBI-SPECT/CT were reviewed prior to inclusion into the study by an expert in the field. The major strength of this study is its prospective head-to-head design with an appropriate power calculation, and the performance of blinded and non-blinded evaluations of the scans by one dedicated nuclear medicine physician and two dedicated radiologists. Patients (*n* = 21) from several hospitals (*n* = 6) were referred for this study when initial imaging (cUS, [^99m^Tc]Tc-MIBI-SPECT/CT, and/or [^18^F]choline PET/CT) failed to localize the parathyroid adenoma. Even in this highly selected group of patients with previous negative imaging, [^11^C]choline PET/CT achieved a high sensitivity of 85%. Moreover, [^11^C]choline PET/CT was able to better localize multi-gland lesions compared with [^11^C]methionine and 4D-CT although in a small patient number.

## Conclusion

This study prospectively compared the diagnostic effectiveness of [^11^C]methionine PET/CT, [^11^C]choline PET/CT, and 4D-CT head-to-head in patients with pHPT after failure of first-line imaging. Our results show that [^11^C]choline PET/CT is superior to [^11^C]methionine PET/CT and 4D-CT in localizing parathyroid adenomas, allowing correct localization in 85% of parathyroid adenomas. Further studies are needed to determine cost–benefit and efficacy of the three studied imaging techniques for the localization of parathyroid adenomas, and to study their feasibility as potential first-line imaging techniques.

### Supplementary Information

Below is the link to the electronic supplementary material.

Supplementary file1 (DOCX 2.55 MB)

## Data Availability

The datasets generated during and/or analyzed during the current study are available from the corresponding author on reasonable request. Dr A. H. B and S. K. had full access to all the data in the study and take responsibility for the integrity of the data and the accuracy of the data analysis.
