# Supplementary material for: Head-to-head comparison of [11C]methionine PET, [11C]choline PET, and 4-dimensional CT as second-line scans for detection of parathyroid adenomas in primary hyperparathyroidism
Source: Eur J Nucl Med Mol Imaging. 2023 Nov 17;51(4):1050–9. doi: 10.1007/s00259-023-06488-7 (PMC10881780; doi:10.1007/s00259-023-06488-7)
Supplement: Supplementary file 1 — Supplementary file1 (DOCX 2.55 MB) [file 259_2023_6488_MOESM1_ESM.docx]

**Head-to-head comparison of [^11^C]methionine PET, [^11^C]choline PET and 4-dimensional CT as second-line scans for detection of parathyroid adenomas in primary hyperparathyroidism**

*European Journal of Nuclear Medicine and Molecular Imaging*

Milou E Noltes1,2,3, Schelto Kruijff1,2,4, Auke P.A. Appelman5, Liesbeth Jansen2, Wouter T Zandee6, Thera P. Links6, Bettien M van Hemel7, Hugo M Schouw1,2, Rudi AJO Dierckx1,3, Anne Brecht Francken8, Wendy Kelder3, Anouk van der Hoorn5, Adrienne H Brouwers1

1 Department of Nuclear Medicine and Molecular Imaging, University Medical Center Groningen, University of Groningen, Groningen, the Netherlands

2 Department of Surgical Oncology, University Medical Center Groningen, University of Groningen, Groningen, the Netherlands

3 Department of Surgery, Martini Hospital Groningen, Groningen, the Netherlands

4 Department of Molecular Medicine and Surgery, Karolinska Institutet, Stockholm, Sweden

5 Department of Radiology, University Medical Center Groningen, University of Groningen, Groningen, the Netherlands

6 Division of Endocrinology, Department of Internal Medicine, University Medical Center Groningen, University of Groningen, Groningen, the Netherlands

7 Department of Pathology, University Medical Center Groningen, University of Groningen, Groningen, the Netherlands

8 Department of Surgical Oncology, Isala, Zwolle, the Netherlands

**Corresponding author**

A.H. Brouwers, MD, PhD

University of Groningen Email: [a.h.brouwers@umcg.nl](mailto:a.h.brouwers@umcg.nl)

University Medical Center Groningen Tel: +31-50-3613541

Department of Nuclear Medicine and Molecular Imaging Fax: +31-50-3611687

Hanzeplein 1, 9700 RB Groningen, The Netherlands

**Methods: First-line imaging techniques**

cUS was performed in the University Medical Center Groningen (UMCG) or referring hospitals on various ultrasound systems by radiologists, as described previously.^1^ The [^99m^Tc]Tc-MIBI-SPECT/CT was performed in the UMCG or the referring hospital. At the UMCG, patients were scanned on a Symbia T16 gamma camera with CT (Siemens), resulting in planar and SPECT/CT images. [^99m^Tc]Tc-MIBI was used for preoperative localization as dual phase technique, combined with a dual tracer subtraction technique for thyroid only visualization with ^99m^Tc-pertechnetate.^1^ [^99m^Tc]Tc-MIBI scans performed in referring hospitals had slightly different imaging protocols (only dual phase technique and/or only SPECT). However, all [^99m^Tc]Tc-MIBI-SPECT/CT were centrally revised in the UMCG, and all referring hospitals adhered to guidelines on parathyroid imaging.^2^

**Methods: [^11^C]methionine PET/CT and [^11^C]choline PET/CT**

[^11^C]methionine PET/CT and [^11^C]choline PET/CT were performed as described before.^3,4^ In short, first, a low dose CT (ldCT) was performed for the attenuation correction of PET images. The ldCT was performed with 100kV, Quel ref mAs of 30 and a pitch of 1 on a 40 or 64-slice CT (Biograph mCT^TM^ or Biograph Vision^TM^, Siemens). Patients had to fast for six hours while drinking one liter of water prior to the PET procedure. PET images were taken 20 minutes after injection of 400 MBq [^11^C]methionine or [^11^C]choline for a duration of 10 minutes in list mode. Scan area involved the area from the lower jaw till upper border of the heart and consisted of one bed position. All images were iteratively reconstructed using three iterations, 21 subsets with 5 mm FWHM Gaussian filter, including time of flight and resolution modelling.

**Methods: 4D-CT**

The 4D-CT parathyroid examinations were performed by using a Dual Source CT scanner (Siemens SOMATOM Force or Siemens SOMATOM Flash, Siemens). The protocol consisted of three imaging phases. The first phase was a nonenhanced CT study to cover the thyroid gland; the z-axis was from the hyoid bone to the clavicular head. The next two phases were contrast-enhanced phases from the angle of the mandible to the carina and were performed after intravenous administration of 70 mL of iomeprol (Iomeron 350 Bracco, Milan, Italy) through a 20-gauge cannula in a right antecubital vein at a rate of 4 mL/sec, followed by a 25-mL saline chaser. Arterial phase images were acquired 25 seconds after the start of the injection and the delayed (venous) phase was acquired 80 seconds from the start of the injection. The acquired images in all three phases were sent to a picture archiving and communication system (PACS, SECTRA) for archiving and also, if necessary, to a three-dimensional workstation to allow for manipulation of images.

**Results: First-line imaging**

All patients (100.0%) had preoperative cUS and [^99m^Tc]Tc-MIBI-SPECT/CT. [^99m^Tc]Tc-MIBI imaging included imaging with [^99m^Tc]Tc-MIBI and [^99m^Tc]-pertechnetate in 14 patients (44%), [^99m^Tc]Tc-MIBI only in 13 patients (41%), [^99m^Tc]Tc-MIBI + [123]I in 4 patients (13%) and [^99m^Tc]-tetrofosmin + [123]I in 1 patient (3%) (Table 1). In 3 patients (9%) only SPECT imaging (without CT) was performed and in 29 patients (91%) SPECT/CT was performed. cUS and [^99m^Tc]Tc-MIBI-SPECT/CT were both negative or inconclusive in 20 patients (63%), cUS was positive and [^99m^Tc]Tc-MIBI-SPECT/CT negative or inconclusive in 11 patients (34%) and cUS was negative and [^99m^Tc]Tc-MIBI-SPECT/CT positive in 1 patient (3%). The patient in whom [^99m^Tc]Tc-MIBI was positive underwent a SPECT/CT with [^99m^Tc]Tc-MIBI only.

| **Table 1. First-line imaging techniques** | |
| --- | --- |
| **Characteristics** | **Total cohort (n=32)** |
| **Imaging techniques** |  |
| cUS and [^99m^Tc]Tc-MIBI imaging, n (%) | 32 (100%) |
| **cUS and hospital** |  |
| At the UMCG, n (%) | 22 (69%) |
| External, n (%) | 10 (31%) |
| **[^99m^Tc]Tc-MIBI and hospital** |  |
| At the UMCG, n (%) | 11 (34%) |
| External, n (%) | 21 (66%) |
| **Tracer technique** |  |
| [^99m^Tc]Tc-MIBI + [^99m^Tc]-pertechnetate, n (%) | 14 (44%) |
| [^99m^Tc]Tc-MIBI only, n (%) | 13 (41%) |
| [^99m^Tc]Tc-MIBI + [123]I, n (%) | 4 (13%) |
| [^99m^Tc]-tetrofosmin + [123]I, n (%) | 1 (3%) |
| **SPECT(/CT)** |  |
| SPECT only, n (%) | 3 (9%) |
| SPECT/CT, n (%) | 29 (91%) |
| cUS= cervical ultrasonography | |

In the blinded scan interpretation of the PET/CT scans, in the patients with both negative cUS and [^99m^Tc]Tc-MIBI-SPECT/CT (n=20 patients), [^11^C]methionine was positive in 17 patients (85%), [^11^C]choline in 19 patients (95%) and 4D-CT in 8 patients (40%), respectively. In the patients with discordant first-line imaging (n=12 patients), [^11^C]methionine was positive in 6 patients (50%), [^11^C]choline in 9 patients (75%) and 4D-CT in 7 patients (58%), respectively.

**Results: Non-blinded scan interpretation**

When the scans were non-blinded from patient data and previous imaging modalities, the number of patients with a positive scan decreased from 23 to 20 for [^11^C]methionine PET/CT, while it increased for [^11^C]choline PET/CT from 28 to 29 patients and for 4D-CT from 15 to 20 patients, respectively (Fig. 1). The number of identified lesions decreased from 23 to 20 for [^11^C]methionine PET/CT and from 32 to 31 for [^11^C]choline PET/CT (Fig. 2), while it increased from 16 to 23 for 4D-CT, respectively. For the overlap between the three imaging techniques, refer to Fig. 3.


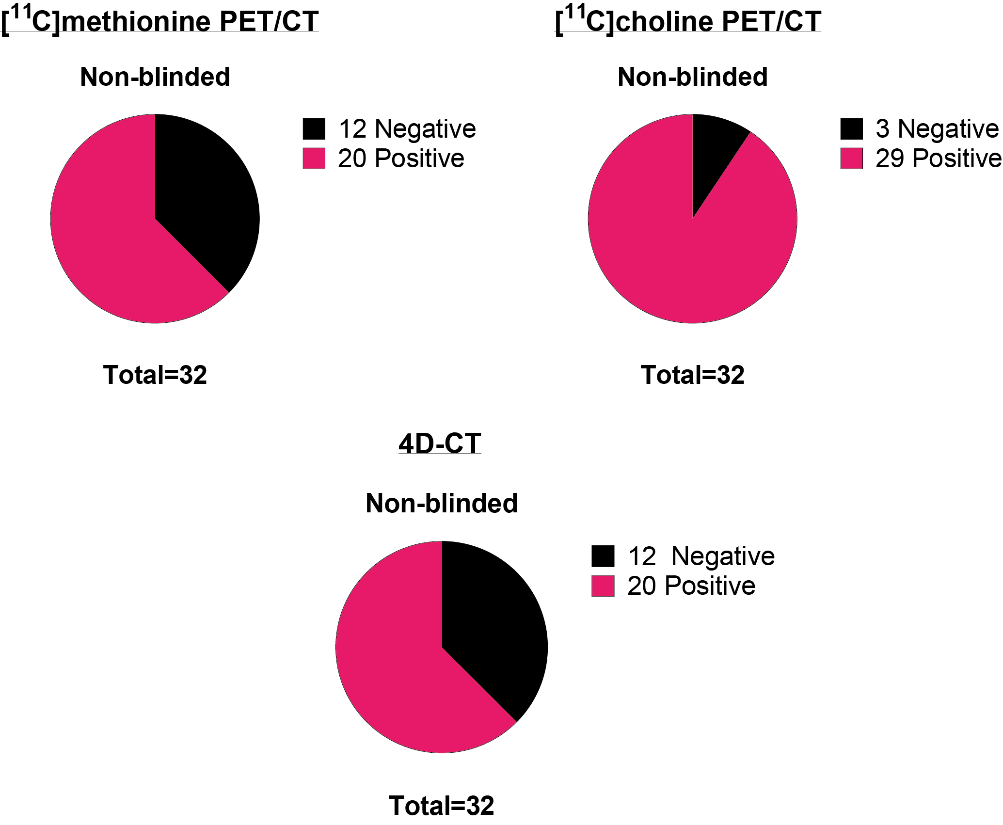


**Fig. 1 Preoperative parathyroid scan interpretation of [^11^C]methionine PET/CT, [^11^C]choline PET/CT, and 4D-CT with non-blinded scan interpretation (patient-based, n=32).**

**Fig. 2 Number of positive lesions and scans for [^11^C]choline PET/CT in the blinded and non-blinded scan interpretation.**


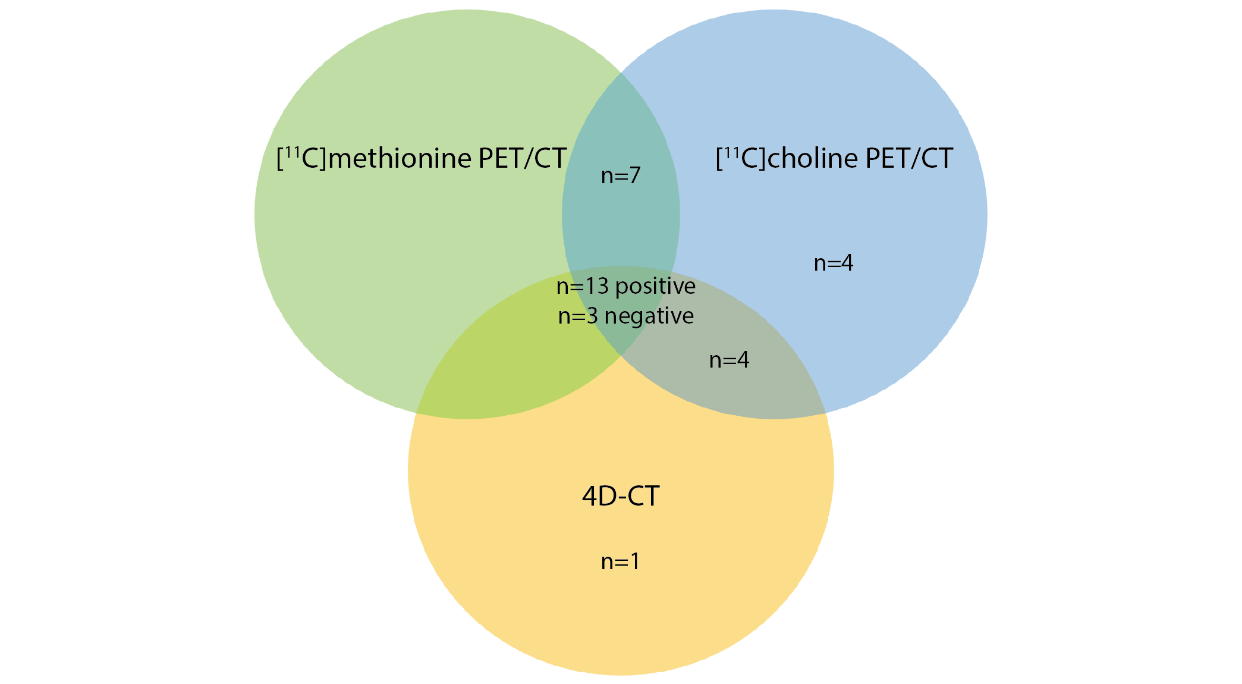


**Fig. 3 Overlap between scan interpretation of [^11^C]methionine** **PET/CT, [^11^C]choline PET/CT, and 4D-CT with non-blinded scan interpretation (patient-based, n=32).** In n=13 patients all scans were positive and n=3 patients all scans were negative.

**Results: Surgery**

Intraoperative PTH (ioPTH) was used in 18/30 patients (60%) and it decreased >50% in 15/18 patients (83%). In the three patients in whom ioPTH did not decrease, in one patient during surgery a parathyroid lesion confirmed as hyperplastic was removed but ioPTH did not decrease sufficiently (27% decrease). As six months after surgery the patient was normocalcemic with high PTH levels (17.50 pmol/L), she was classified as cured.[31] In the second patient, two parathyroid glands were surgically removed and confirmed as hyperplasia at pathological examination. Six months after surgery the patient was still hypercalcemic and four-gland hyperplasia was suspected. In the third patient, PTH levels were taken prior to removal of a thyroid nodule. On the [^11^C]choline PET/CT, the suspected lesions was located intra-thyroidally. After removal of wat the surgeon initially suspected might be the parathyroid adenoma, ioPTH was performed. When it did not decrease sufficiently, the surgeon decided to remove the thyroid nodule. At pathological examination, the adenoma was located intra-thyroidally and the patient was normocalcemic on postoperative day one and six months after surgery.

**Results: Blinded accuracy of study imaging modalities**

For the overlap between scan interpretation and histological results with blinded scan interpretation, refer to Fig. 4.

**Fig. 4 Overlap between scan interpretation and histological results of [^11^C]methionine PET/CT, [^11^C]choline PET/CT, and 4D-CT with blinded scan interpretation (patient-based, n=30).** TP= true positive, FN= false negative.

**Results: Non-blinded accuracy of study imaging modalities**

In the patients with multi-gland disease (n=3 patients and n=6 lesions), [^11^C]methionine PET/CT was true positive in 1 lesion (17%), while [^11^C]choline PET/CT and 4D-CT were true positive in 3 lesions (50%) at the non-blinded scan interpretation. For the results of the effectiveness of the studied imaging modalities, refer to Fig. 5.

The sensitivity of [^11^C]choline PET/CT differed significantly from [^11^C]methionine PET/CT and 4D-CT for the non-blinded scan interpretations (p=0.004 and p=0.001, respectively) (Table 2). The sensitivity of [^11^C]methionine PET/CT and 4D-CT did not differ significantly for the non-blinded interpretations (p=0.774).


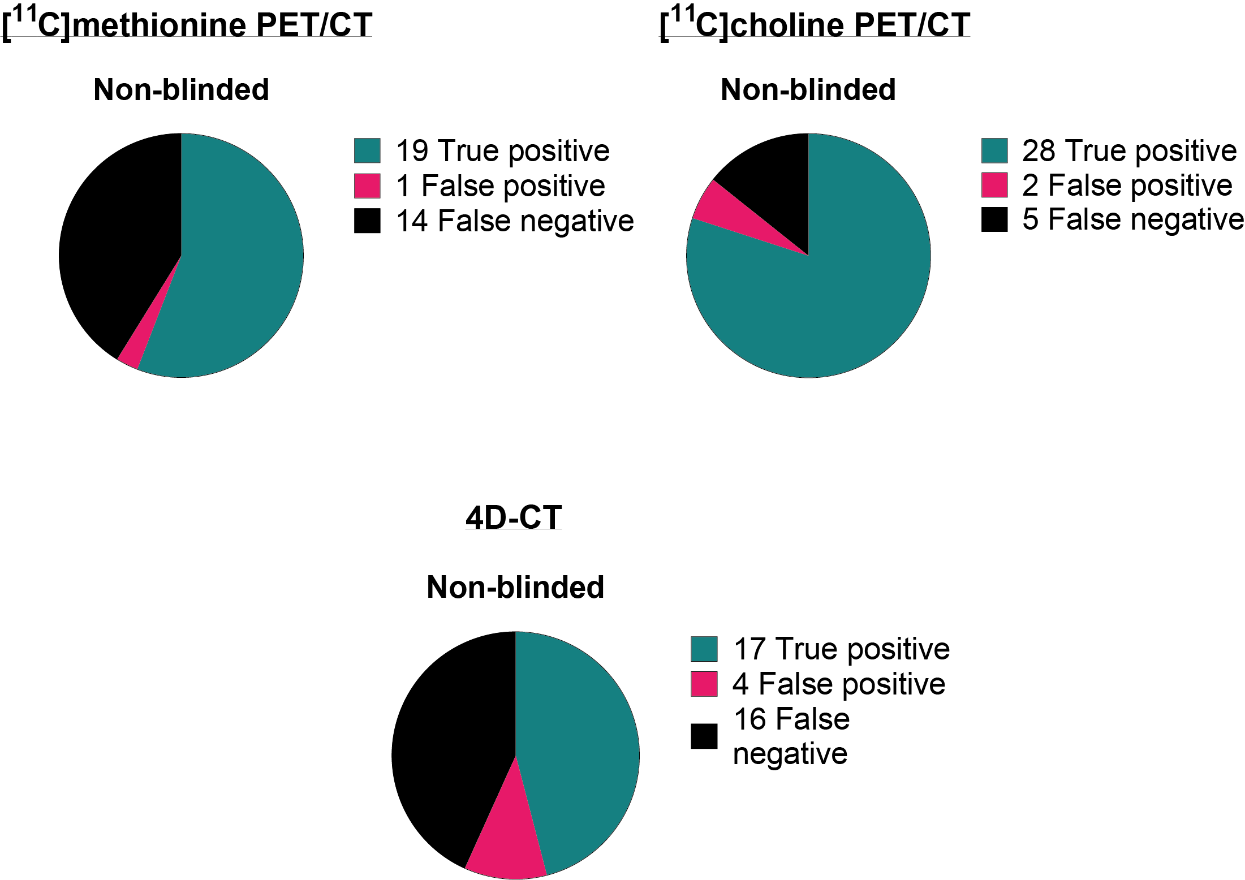


**Fig. 5 Effectiveness of the three studied imaging modalities for parathyroid adenoma localization with non-blinded scan interpretation (lesion-based).** Adenoma localization on preoperative imaging was correlated with intraoperative surgeon localization and histopathological findings (reference standard). The total number of reference standard lesions (true positive + false negative) consists of n=32 pathology-proven parathyroid lesions and one patient with a negative exploration (n=1 lesion) in n=30 operated patients.

| **Table 2**. Final lesion-based outcome with non-blinded scan interpretation for the n=30 operated patients*. | | | | | |
| --- | --- | --- | --- | --- | --- |
|  | **Non-blinded scan interpretation** | | | | |
|  | **True positive** | **False positive** | **False negative** | **Sensitivity**  **(95% CI)** | **PPV**  **(95% CI)** |
| [^11^C]methionine PET/CT | 19 | 1 | 14 | 58%  (39–75%) | 95%  (73–100%) |
| [^11^C]choline PET/CT | 28 | 2 | 5 | 85%  (68–95%) | 93%  (77–99%) |
| 4D-CT | 17 | 4 | 16 | 52%  (34–69%) | 81%  (57–94%) |
| *CI=* confidence interval; *PPV=* positive predictive value; *= total consists of 32 pathology-proven parathyroid lesions and one patient with a negative exploration. | | | | | |

**Results: Case examples of patients with a negative [^18^F]choline PET/CT**

Figure 6 displays an example of a case with a previous negative [^18^F]choline PET/CT. This patient was referred to our clinic due to an inconclusive [^99m^Tc]Tc-MIBI-SPECT/CT and negative [^18^F]choline PET/CT, while cUS showed an inconclusive lesion on the dorsal-caudal side of the left thyroid lobe. After inclusion into the study, [^11^C]choline PET/CT and 4D-CT both showed two parathyroid lesions at the non-blinded scan interpretation: one on the caudal-lateral side of the left thyroid lobe and one on the dorsal-caudal side of the right thyroid lobe, while [^11^C]methionine was negative. At the blinded scan interpretation, [^11^C]choline PET/CT identified the same two lesions, however 4D-CT only identified one of the two lesions. During surgery the parathyroid lesion on the caudal-lateral left side was first removed and ioPTH was performed. After 20 minutes, the ioPTH decreased by only 44% (from 8.9 pmol/L to 5.0 pmol/L). Due to an insufficient decrease in PTH (<50%), the right side of the neck was also explored and a parathyroid lesion on the dorsal-caudal side was removed, after which the ioPTH decreased to 30% (2.7 pmol/L). Pathology showed two hyperplastic parathyroid glands (0.4 gram and 11 mm in diameter and 0.17 gram and 9 mm in diameter, respectively), resulting in two correct positive lesions for [^11^C]choline PET/CT and 4D-CT and two false negative lesions for [^11^C]methionine PET/CT from the non-blinded interpretation. One year after the surgery, the patient remained normocalcemic.

Another representative case is depicted in Figure 7. In this patient, the [^99m^Tc]Tc-MIBI-SPECT/CT, cUS and [^18^F]choline PET/CT were all negative. Subsequently, the patient was included in the study and at the blinded scan interpretation [^11^C]methionine PET/CT revealed a parathyroid lesion cranial to the left thyroid lobe, while [^11^C]choline PET/CT showed two parathyroid lesions (cranial and caudal to the left thyroid lobe) and 4D-CT was negative. At the non-blinded scan interpretation, both [^11^C]methionine PET/CT, [^11^C]choline PET/CT and 4D-CT revealed one parathyroid lesion cranial to the left thyroid lobe. During surgery two hyperplastic parathyroid glands (0.43 gram and 13 mm, and 0.28 gram and 12 mm) cranially and caudally to the left thyroid lobe were extirpated, resulting in one correct positive and one false negative lesion for all scans at the non-blinded interpretation. At the blinded interpretation, [^11^C]choline PET/CT scored two correct positive lesions. Eight months after surgery, the patient remained normocalcemic.

***
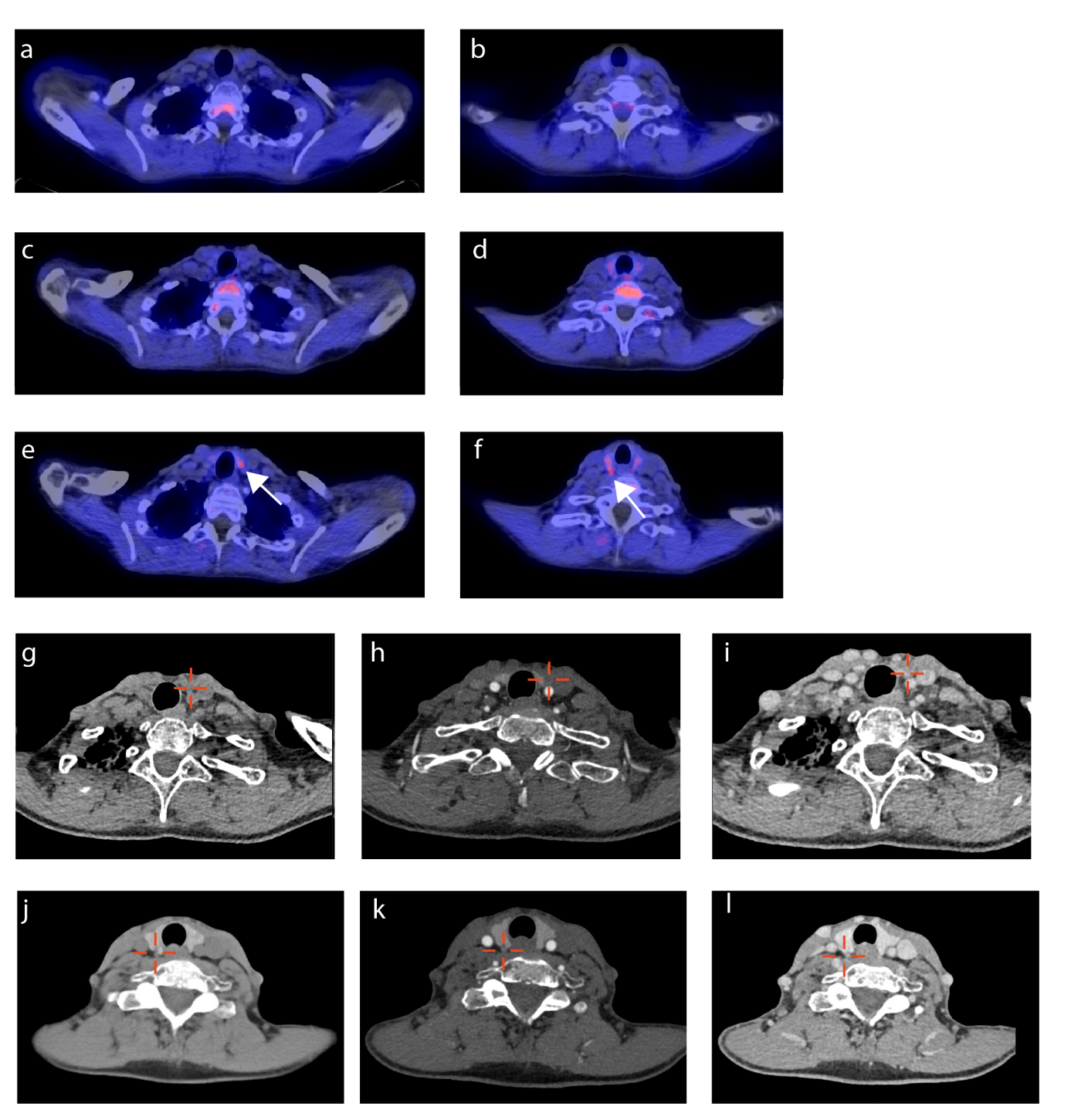
***

**Fig. 6 A representative patient referred to our study with a negative [^18^F]choline** **PET/CT and two parathyroid adenomas.** The [^18^F]choline PET/CT (**a, b** fused PET/CT images) and [^11^C]methionine PET/CT (**c, d** fused PET/CT images) did not show a parathyroid adenoma. On the [^11^C]choline PET/CT (**e, f** fused PET/CT images) and 4D-CT (**f/j** nonenhanced phase**, h/k** arterial phase, **i/l** venous phase) two parathyroid adenomas were identified at the non-blinded interpretation (on the caudal-lateral side of the left thyroid lobe and on the dorsal-caudal side of the right thyroid lobe, indicated with the white arrow and delineated in red). Both were removed during parathyroidectomy and at pathology confirmed to be hyperplastic parathyroid glands.


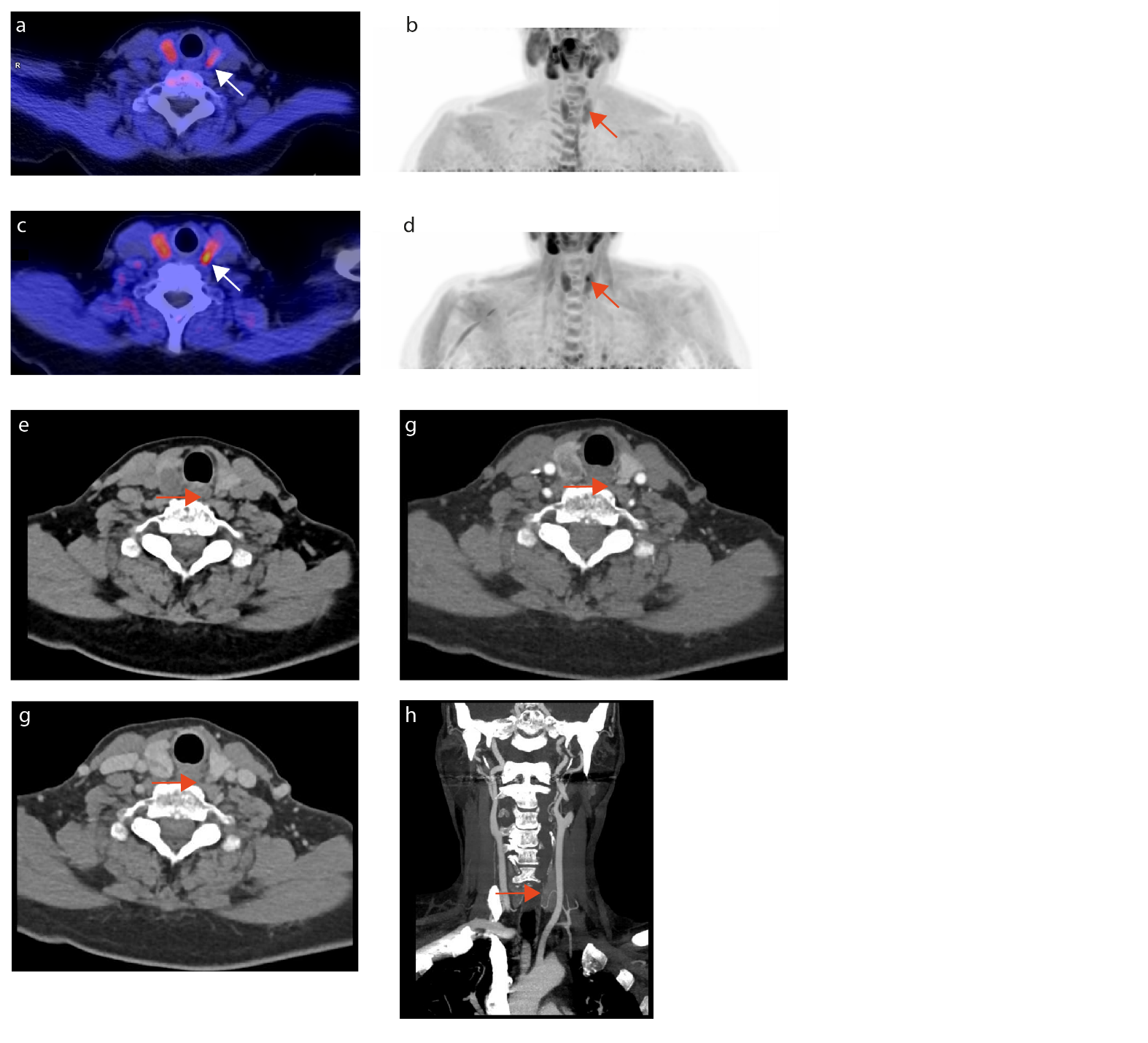


**Fig. 7 A patient with two parathyroid adenomas referred to our study with a negative [^18^F]choline PET/CT.** On the [^11^C]methionine PET/CT (**a, b**; weakly positive), [^11^C]choline PET/CT (**c, d**; strongly positive) (**a/c** fused PET/CT image and **b/d** maximum intensity projection) and 4D-CT (**e** nonenhanced phase**, f** arterial phase, **g** venous phase) a parathyroid adenoma was shown cranially to the left thyroid lobe (white and red arrows). The 4D-CT identified a prominent inferior thyroid artery going to the parathyroid lesion (**h**).

**References**

1. Noltes M, Coester A, van der Horst-Schrivers A, et al. Localization of parathyroid adenomas using 11C-methionine pet after prior inconclusive imaging. *Langenbecks Arch Surg*. 2017;402:1109-1117.
2. Ovčariček P, Giovanella L, Gasset I, et al. The EANM practice guidelines for parathyroid imaging. *Eur J Nucl Med Mol Imaging.* 2021;48:2801-2822.
3. Noltes M, Coester A, van der Horst-Schrivers A, et al. Localization of parathyroid adenomas using 11C-methionine pet after prior inconclusive imaging. *Langenbecks Arch Surg*. 2017;402:1109-1117.
4. Noltes ME, Kruijff S, Jansen L, et al. A retrospective analysis of the diagnostic performance of (11)C-choline PET/CT for detection of hyperfunctioning parathyroid glands after prior negative or discordant imaging in primary hyperparathyroidism*. EJNMMI Res*. 2021;11(1):32-7. doi: 10.1186/s13550-021-00778-7 [doi].
